# A Personal History of Cystinosis by Dr. Jerry Schneider

**DOI:** 10.3390/cells11060945

**Published:** 2022-03-10

**Authors:** Jerry Schneider, Elena Levtchenko

**Affiliations:** 1 Department of Pediatrics, School of Medicine, University of California San Diego, La Jolla, CA 92093, USA; jschneider@ucsd.edu; 2 Department of Development and Regeneration, Katholieke Universiteit (KU) Leuven, 3000 Leuven, Belgium; 3 Department of Paediatrics, Division of Nephrology, University Hospitals Leuven, 3000 Leuven, Belgium

**Keywords:** cystinosis, lysosomal storage disorder, history, treatment strategies for cystinosis

## Abstract

Cystinosis is a rare lysosomal storage disease that is tightly linked with the name of the American physician and scientist Dr. Jerry Schneider. Dr. Schneider (1937–2021) received his medical degree from Northwestern University, followed by a pediatrics residency at Johns Hopkins University and a fellowship in inherited disorders of metabolism. He started to work on cystinosis in J. Seegmiller’s laboratory at the National Institutes of Health (NIH) and subsequently moved to the UC San Diego School of Medicine, where he devoted his entire career to people suffering from this devastating lysosomal storage disorder. In 1967, Dr. Schneider’s seminal *Science* paper ‘Increased cystine in leukocytes from individuals homozygous and heterozygous for cystinosis’ opened a new era of research towards understanding the pathogenesis and finding treatments for cystinosis patients. His tremendous contribution transformed cystinosis from a fatal disorder of childhood to a treatable chronic disease, with a new generation of cystinosis patients being now in their 40th and 50th years. Dr. Schneider wrote a fascinating ‘Personal History of Cystinosis’ highlighting the major milestones of cystinosis research. Unfortunately, he passed away before this manuscript could be published. Fifty-five years after his first paper on cystinosis, the ‘Personal History of Cystinosis’ by Dr. Schneider is a tribute to his pioneering discoveries in the field and an inspiration for young doctors and scientists who have taken over the torch of cystinosis research towards finding a cure for cystinosis.

## 1. Introduction

When people write about the history of cystinosis, they usually start with Emil Abderhalden, who in 1903 described cystine crystals in the liver and spleen at the autopsy of a 21-month-old child [1]. For the next 30 years, physicians reported occasional cases of “cystine storage diseases” in children. It is not possible to know which of these patients actually had cystinosis because of the confusion between cystinosis and cystinuria at that time.

In cystinosis, cystine accumulates within cells [2], while in cystinuria, the cystine content of cells is normal, but the cystine concentration is elevated in the urine to such a degree that cystine can come out of solution and form cystine stones within the urinary tract [3]. Cystine was first discovered in renal stones from patients with cystinuria. These stones were found to be a unique compound by a British doctor, William Hyde Wollaston, who first called them “cystic oxide”, cystic because the word cyst sometimes referred to the bladder in those days and oxide because Wollaston guessed they contained oxygen [4]. In fact, they did not contain oxygen, and the name was changed to cystine. Cystinuria is about ten times more common than cystinosis, but because the names of these conditions are so similar, people often get them confused.

## 2. Unravelling the Cellular Basis of Cystinosis

I saw my first cystinosis patient in 1965 at the National Institutes of Health (NIH) where I spent two years studying cystinosis in J. Seegmiller’s laboratory. At that time, we knew that cystinosis patients had cystine crystals in most of their tissues. They also had delayed growth, very large urine volume, developed rickets and excreted excessive glucose and protein in their urine. We also knew it was an inherited disorder, but no one knew its cause. These patients had normal intelligence and, if treated symptomatically, would do relatively well for several years. Symptomatic treatment meant replacing orally what they lost in their urine. However, by 9–10 years of age, they were in end-stage kidney failure. At this time, neither renal dialysis nor transplantation was available for them. Since then, much has been learned about cystinosis, both the basic defect that causes this disorder and its treatment. I believe that each advance in our understanding of cystinosis is always followed new technology, often developed by scientists who have never heard of this disease. I will also discuss why it repeatedly took a long time to evaluate new potential treatments.

Early investigators had been stymied by the lack of a good method to measure cystine. I was very lucky that ion-exchange chromatography had just been developed to measure small amounts of amino acids [5]. Furthermore, there was an amino acid analyzer at the NIH that I could use. A visiting scientist at the NIH, John Crawhall, had just developed a better method to measure cystine with this analyzer and was eager to help me [6]. In addition, the company that manufactured this analyzer had just increased its sensitivity ten-fold. Without all of this help, my studies never would have succeeded.

**Figure 1:**
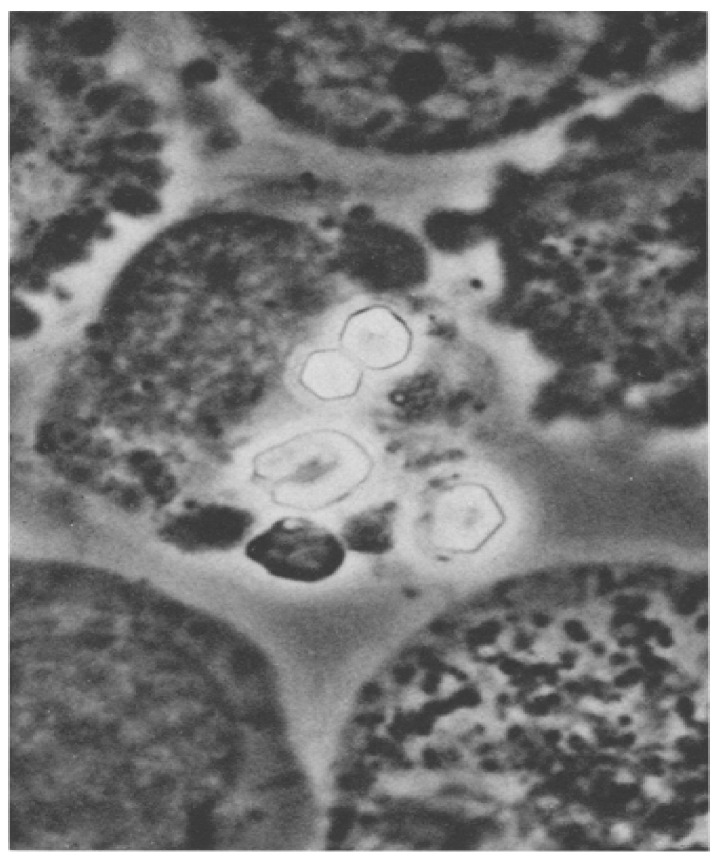
Cystine crystals seen in an unstained bone marrow aspirate obtained from a child at 10 weeks of age. (Phase Optics. ×1280) [6].

When I looked at bone-marrow aspirates from cystinosis patients, I saw huge clumps of cystine crystals (Figure 1) [6]. I wondered if the crystals were inside or outside the cells. If the crystals were inside the cells, was the cystine concentration so great in these cells that the crystals formed intracellularly, or had the crystals formed extracellularly and been phagocytized (ingested) by the cells?

I also found that the solubility of cystine in human plasma at 37° and at physiological pH was about 50 times higher than the plasma cystine concentration in cystinosis patients. This proved to me that the cystine crystals must have formed intracellularly. I later found that a pathologist in Birmingham, England had come to the same conclusion by careful study of pathological sections from cystinosis patients.

I isolated leukocytes in small volumes of blood from cystinosis patients and found that the amount of soluble cystine in these cells was 50–100 times higher than in leukocytes isolated from individuals who did not have cystinosis [7]. We also performed the same measurements in cultured fibroblasts grown from skin biopsies of cystinosis patients and from normal controls. Although we never saw cystine crystals in cystinotic fibroblasts, once again the cystine content was 50–100 times higher in cells from cystinosis patients than in non-cystinotic control cells [8].

My two-year assignment in Seegmiller’s lab was coming to an end and I performed one more experiment that excited me. I wondered if there was an enzyme that reduced cystine to cysteine (“half-cystine”) that might be defective in cystinotic tissue. I decided to develop an essay for glutathione reductase and compare the activity of this enzyme in cystinotic compared to normal tissue. The idea was that glutathione reductase might indirectly reduce cystine to cysteine. In normal tissue, the cystine would be reduced to cysteine and be further metabolized, whereas in cystinotic tissue, the cystine would not be metabolized and would accumulate and form crystals. Sure enough, I found no glutathione reductase activity in the cystinotic tissue, but high amounts of glutathione reductase activity in the normal control tissue. I was very excited by this finding; fortunately, I decided to conduct control studies. I added cystine to normal tissue, and the activity of glutathione reductase disappeared. I dialyzed the cystinotic tissue for a long period with large volumes of solution to remove the cystine, and the activity of this enzyme returned to normal. It was obvious that cystine inhibited the activity of glutathione reductase. Some reading in the library informed me that cystine inhibits many common intracellular enzymes that are necessary for the normal life of cells. Just before leaving the NIH for further training in other areas of science, I conducted a brief experiment that demonstrated that cystine was somehow compartmentalized in the cell so that it did not inhibit the activity of important cellular enzymes.

The next physician who studied cystinosis in Seegmiller’s laboratory was Joe Schulman. He found that the excess cystine in cystinotic tissue was located in the lysosomes of the cells. The Belgian scientist Christian de Duve shared the 1974 Nobel Prize in Physiology or Medicine for his discovery of lysosomes, an intracellular organelle that “digests” proteins and other cellular molecules [9]. Another Belgian investigator, Henri Géry Hers, later found that a defective lysosomal enzyme was the cause of a rare condition called Pompe Disease [10]. We now know that many other rare diseases result from the lack of a specific lysosomal enzyme. However, no lysosomal enzyme is known to metabolize cystine. At this point, we knew that cystine accumulated in cystinotic lysosomes, presumable from protein degradation, but did not understand why it accumulated in cystinotic and not in normal lysosomes.

Seegmiller moved to the University of California San Diego (UCSD) in 1969, and Schulman took over his NIH laboratory. In 1970, Seegmiller recruited me to UCSD, where I was able to establish my own laboratory in the Department of Pediatrics. Schulman and I were friends and decided we would both continue to study cystinosis. I decided the major problem we had in these studies was how time consuming and difficult it was to assay cystine. Since each assay took 8 h, we could only perform three cystine assays a day on our one amino acid analyzer (I had to come to the laboratory each evening at midnight to start the third measurement.) Fortunately, I learned that a biochemist (Clement Furlong) at the University of California Riverside was isolating proteins from the bacteria E. coli that had the property of specifically binding small molecules and that there was a cystine-binding protein. I thought this might allow us to develop a sensitive assay for cystine. I called Furlong and asked if he could help me isolate this protein. He told me he was preparing some of these proteins later that week and he thought he knew where the cystine-binding protein came off the column he used to prepare these proteins. He invited me to visit and take some of this material. I had a new post-doctoral fellow (Robert Oshima) who had just arrived and we drove to Riverside later that week (90 min drive). We returned that evening with a beaker full of solution that we hoped contained the cystine-binding protein. I went home for dinner and sleep. Oshima was too excited to sleep and worked all night with the material we obtained and the next morning told me he was certain it would work. We submitted a paper to the Journal of Biological Chemistry two months later describing the use of this cystine-binding protein to measure small amounts of cystine [11]. For many years this assay was the “gold standard” for measuring intracellular cystine.

## 3. Discovery of Cysteamine Treatment 

Before I continue describing our laboratory studies, I must discuss some of the attempts to find a specific treatment for cystinosis. It is never easy to find a successful treatment for any disease, but in some cases it quickly becomes obvious that the treatment works. Of course, you have to be certain the treatment is not harmful, but at least you know if it works. For instance, if you develop an antibiotic for a certain bacterial infection, you soon learn if the antibiotic kills that bacteria. Cystinosis is much more difficult. The kidney damage we were attempting to arrest progresses very slowly, and our methods of measuring kidney function in children were not very exacting. We assumed that the cystine was causing the kidney damage, but we could not be certain. It was always possible that whatever was causing the storage of cystine was also causing the kidney damage, and that the two processes were not related. Fortunately, this was not to be the case, and measurement of leukocyte cystine proved to be an excellent marker of whether any particular drug was effective in treating cystinosis.

Over the years many approaches were tried to specifically treat cystinosis and they all seemed to work! I will discuss two treatments that did not work and one that did. Horst Bickel directed the first treatment I will discuss. Dr. Bickel trained in pediatrics from 1947 to 1949 at the University Children’s Hospital in Zurich with Professor Fanconi. He then worked as a research fellow at the University Children’s Hospital in Birmingham, England from 1949 to 1954. While in Birmingham, Bickel developed the successful treatment for another inherited metabolic disease, phenylketonuria (PKU). This condition was treated with a specific diet very low in the amino acid phenylalanine [12]. It was reasonable for Dr. Bickel to attempt a diet low in cystine and other sulfur-containing substances for the treatment of cystinosis. Initially, this diet seemed so successful that it was made and distributed by a major company. The children felt better, their schoolteachers and parents thought they were better, etc. I was still working at the NIH and started several cystinosis patients on this diet. I found that their plasma cystine concentration, which was normal to start, decreased significantly, but their leukocyte cystine content remained elevated. Years later, Dr. Bickel obtained data on all the patients who received this diet and learned that they had died sooner than expected. Apparently, the diet caused liver damage. The fact that they all felt the diet was working is called a “placebo effect”.

Our group tried another treatment that did not work. A German post-doctoral fellow working in my lab (Wolfgang Kroll) found that adding fresh ascorbic acid (Vitamin C) to the culture media in which cystinotic fibroblasts were growing lowered the cystine content of the cells by over 50%. He was very eager to publish this finding. I thought we should try to understand why this occurred before submitting a paper for publication. Dr. Kroll was relentless in insisting we publish the finding even though we did not understand it. Furthermore, he wanted to submit it to the very prestigious journal, *Science*. I finally agreed to submit the paper being certain it would be promptly rejected and Dr. Kroll would learn to trust his mentor. To my astonishment, the paper was promptly accepted [13]. I was concerned that when this publication appeared every cystinotic patient in the world would be started on a high dose of Vitamin C, and it would take years before we knew if it worked. I promptly organized a placebo controlled, masked study. Sixty-four patients entered the study; half received high doses of Vitamin C and half received a placebo. They all were certain they were receiving the actual drug because they all felt better. They saw their physicians every 4 months for routine tests of kidney function, and the results were sent to a committee at the NIH who knew which patients were receiving the drug and which the placebo. After about 18 months, I received an emergency phone call from the committee telling me to stop the study at once because the patients receiving a high dose of Vitamin C were doing worse than the patients receiving the placebo. This study was published in the New England Journal of Medicine [14].

**Figure 2:**
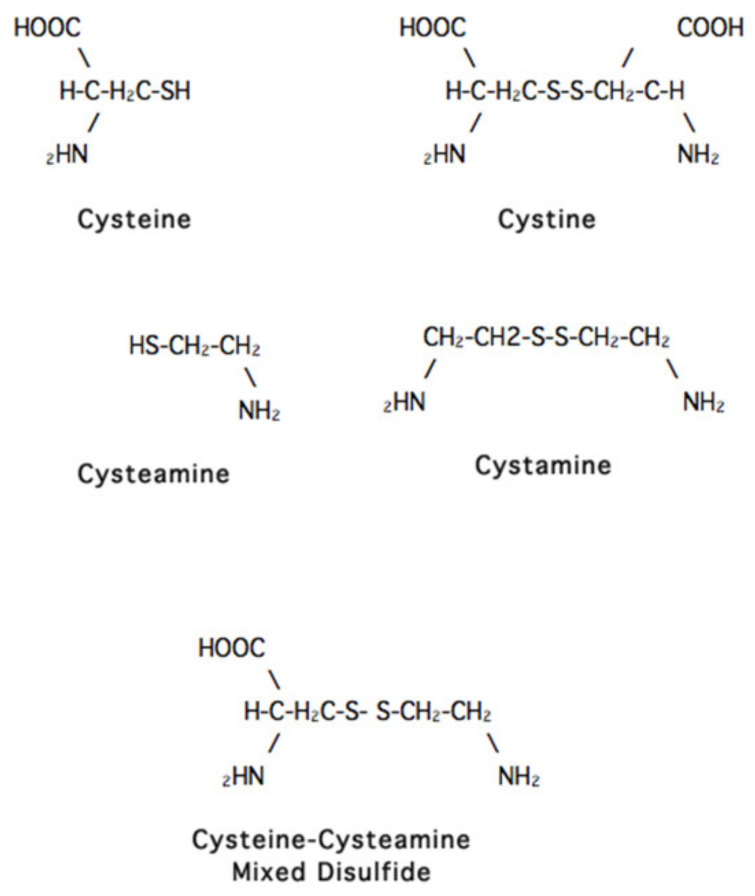
Structures of compounds mentioned in this article. The two compounds shown on the upper left are sulfhydryls because they have a -SH group. The compounds to their right are disulfides. The compound at the bottom is a mixed disulfide of two different compounds.

John Crawhall, the scientist who helped me at the NIH, began the study that did work. He was then working in Montreal but came to UCSD for a sabbatical and decided to add a variety of compounds to the media in which cystinotic cells were growing to see if any had an effect on the cystine content of these cells. The only compound that seemed to have such an effect was cysteamine (Figure 2). By the time we saw this result, Crawhall had returned to Montreal, and I assigned the project to a new post-doctoral fellow, Jess Thoene.

Cysteamine occurs naturally in humans, but at very low concentrations [15]. It was being used successfully in Europe as an antidote for an overdose of acetaminophen (called paracetamol in Europe and usually Tylenol in the USA). Experiments were also being conducted for its use as a radio-protective agent. Thoene found that cysteamine not only removed cystine from cystinotic cells in culture, but also removed the cystine from leukocytes in a cystinosis patient when given either intravenously or orally [16]. He worked out the proper dose to use orally, and this is essentially the same dose used today.

We joined with the NIH group of Joe Schulman, his post-doctoral fellow Bill Gahl and biostatistician James Schlesselman to start a large study. Many physicians contributed patients, including Michel Broyer in Paris and William van’t Hoff in London. In 1987, we reported the success of cysteamine in treating cystinosis [17]. At first, we used cysteamine hydrochloride. It had to be distributed as a liquid and had a horrible smell and taste. We next used phosphocysteamine. In its pure form it was odorless, but we could not obtain this chemical in the pure form in the quantities required. It was somewhat better than cysteamine hydrochloride, but still difficult for patients to take and could not be manufactured in a way that regulatory agencies would ever approve.

## 4. Cystinosis as a Treatable Lysosomal Storage Disease

**Figure 3:**
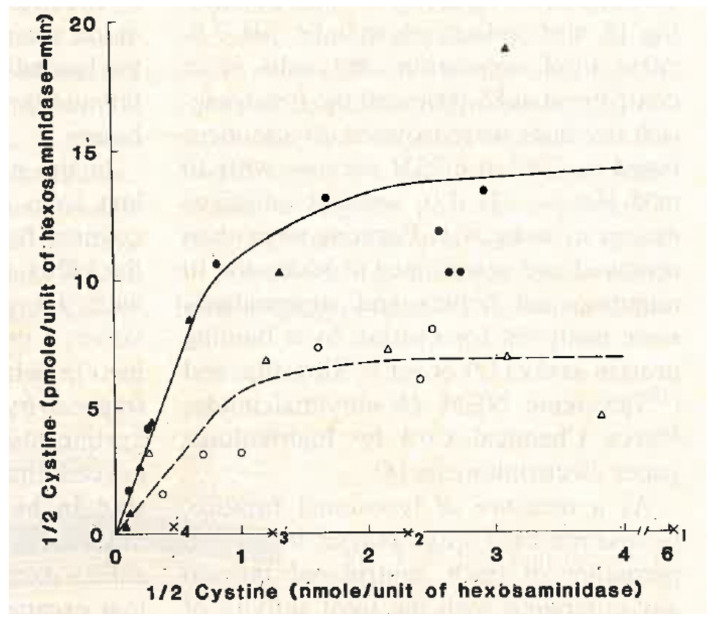
Lysosomes from cystinosis patients (x), their parents (open symbols) and normal controls (filled symbols) were loaded with cystine, and the initial velocity of cystine egress was compared [20].

Meanwhile, we continued to search for the metabolic defect in cystinosis. We all agreed that the most likely cause was a transport defect in the lysosomal membrane. As proteins are digested in the lysosomes, individual amino acids are released. Specific transporters exist in the lysosomal membrane that carry specific amino acids from the lysosome to the cytoplasm of the cell. Could the transporter for cystine be missing in cystinosis lysosomes? None of us knew how to test this idea. Eric Harms visited our laboratory from Germany and helped us isolate excellent preparations of lysosomes, but we still did not know how to study transport in the lysosomes. The breakthrough came when Frank Tietze, a biochemist at the NIH, saw a paper in the Journal of Biological Chemistry and pointed it out to Drs. Schulman and Gahl at the NIH. This paper described a method of loading lysosomes with a specific amino acid [18]. Thus, we could study the egress of this amino acid from the lysosome. Dr. Schulman pointed out this paper to my post-graduate fellow, Adam Jonas, and me. We decided that the NIH group would study this method in cystinotic leukocytes, and the UCSD group would use cultured cystinotic fibroblasts. At UCSD, we were greatly helped by the contributions of Drs. Margaret Smith and Alice Greene. Both groups found that the lysosomal transport of cystinosis was defective in cystinotic cells (Figure 3) [19,20].

The groups at the NIH and the University of Michigan (Jess Thoene, Halvor Christensen and Ron Pisoni) found that cysteamine works in the cystinotic lysosome by “attacking” the cystine molecule and forming a “mixed disulfide” of half cystine and cysteamine. This “mixed disulfide” resembles the amino acid lysine and exits the lysosome via the alternative transporting system that is normal in cystinotic lysosomes [21]. Finally, Corinne Antignac’s group in Paris found that the defective gene in cystinosis is located on chromosome 17 and encodes the lysosomal cystine transporter cystinosin [22]. 

In the 1980s, we began to use cysteamine bitartrate to treat cystinosis. Although this drug is also difficult to synthesize, a company in Italy was producing it. The drug was stable as a solid and more acceptable for patients. In 1994, the United States Food and Drug Administration (FDA) approved cysteamine bitartrate for the treatment of cystinosis. Although this formulation of cysteamine was very effective in removing cystine from cystinotic cells, it still had several problems as a drug for cystinosis. It has a very bad smell and taste, and must be taken every 6 h to be completely effective. Elena Levtchenko, in Belgium, showed that if a patient went 8 h between doses, for instance while sleeping, the leukocyte cystine content went unacceptably high in the last two hours [23]. For acceptable treatment, neither the patient nor his/her caregivers ever had a full night sleep. A pediatric gastroenterologist Ranjan Dohil performed careful studies of cysteamine uptake by the intestine and developed a way to package cysteamine so that it would be easier for patients to take and would only have to be taken every 12 h [24]. Recently, the FDA and EMA approved this new extended-release form of cysteamine.

If started early in life, cysteamine bitartrate slows the progression of kidney glomerular but not kidney tubular damage. Studies from both the NIH and Hôpital Necker Enfants-Malades in Paris found that, even if started at an older age, cysteamine delays the progression of hypothyroidism, diabetes and neuromuscular disorders [25,26]. However, this is not a cure for cystinosis. Stephanie Cherqui, one of Antignac’s students, moved to San Diego, and using a mouse model of cystinosis that she had developed in Antignac’s laboratory, used hematopoietic stem cell (HSC) gene therapy to essentially correct the cystinosis defect in the mouse organs [27]. Currently, a clinical trial is underway to test the effect of gene-corrected autologous HSC in cystinosis patients [28].

## 5. Conclusions

Jerry Schneider devoted his whole career to unraveling the disease mechanisms and finding a curative therapy for cystinosis. His work transformed cystinosis from a fatal condition of childhood into a treatable disease with which patients can now reach advanced adult age. Dr. Schneider inspired several generations of clinicians and researchers to take over the torch of cystinosis research and to find cure for this devastating disorder. Fifty fine years after the first pioneering paper on cystinosis in *Science* 1967, the Special Issue of Cells is devoted to cystinosis and to the tremendous contribution of Dr. Schneider as a pioneer and founder of cystinosis research.
